# Longitudinal study of dental caries incidence associated with *Streptococcus mutans* and *Streptococcus sobrinus* in patients with intellectual disabilities

**DOI:** 10.1186/s12903-015-0087-6

**Published:** 2015-09-02

**Authors:** Yuki Oda, Fumiko Hayashi, Mitsugi Okada

**Affiliations:** Department of Special Care Dentistry, Hiroshima University Hospital, 1-2-3, Kasumi, Minami-ku, Hiroshima, 734-8553 Japan

## Abstract

**Background:**

Mutans streptococci (*Streptococcus mutans* and *S. sobrinus*) are considered to be major etiologic agents of dental caries. Using a polymerase chain reaction method, we detected those bacteria from 145 outpatients (6–30 years old) with intellectual disabilities (ID) and their presence was compared with the incidence of dental caries.

**Methods:**

Plaque samples were collected from all erupted tooth sites in subjects with a sterile toothbrush. A dental examination was performed to determine the number of decayed and filled teeth (DFT score) in permanent dentition using the WHO caries diagnostic criteria. A Mann–Whitney U-test was employed to compare the caries scores between combinations of the bacteria, and with a Wilcoxon rank test used to compare caries scores between the baseline and after 1 year.

**Results:**

Among all subjects, *S. mutans* and *S. sobrinus* were possessed by 78.7 and 83.5 %, respectively, while 13.1 % were positive for *S. mutans* alone, 17.9 % for *S. sobrinus* alone, and 65.6 % for both organisms, with 3.4 % were negative for both. The mean DFT score of subjects positive for both *S. mutans* and *S. sobrinus* at after 1 year was significantly higher than that of those positive for *S. mutans* alone (*P* < 0.01). The increase in caries increment was also significantly greater in subjects with both bacteria detected (*P* < 0.001).

**Conclusion:**

Our results indicate that patients with ID harboring both *S. mutans* and *S. sobrinus* have a significantly higher incidence of dental caries than those with *S. mutans* alone.

## Background

Mutans streptococci (*Streptococcus mutans* and *S. sobrinus*) are considered to be major etiologic agents of dental caries in humans [1–3]. These bacteria are the most common putative pathogens isolated from human dental plaque and their prevalence has been reported in epidemiological studies [4–7]. Various methods have been used for the detection of putative pathogens, including direct microscopy, cultivation, enzyme tests, enzyme-linked immunosorbent assays and species-specific DNA probes. Several investigators have also developed polymerase chain reaction (PCR) methods and reported them to be more sensitive for detection as compared to conventional culture techniques [8, 9], as they have been shown to be capable of detecting low numbers (5–100) of bacterial cells [9, 10], while they are also quick and relatively simple to perform. Furthermore, PCR assays have been found suitable for specific detection and identification of human cariogenic bacteria, including *S. mutans* and *S. sobrinus* [10, 11].

In previous cross-sectional and longitudinal studies, we reported that preschool children with primary dentition harboring both *S. mutans* and *S. sobrinus* had a significantly higher incidence of dental caries than those with *S. mutans* alone [6, 12]. Recently, we also noted that schoolchildren harboring both *S. mutans* and *S. sobrinus* had a significantly greater dental caries experience in both permanent and primary teeth as compared to those with *S. mutans* alone [13]. Thus, identification and determination of the prevalence of those pathogens are of fundamental importance for understanding the initiation and development of the dental caries, and for determining better forms of treatment and prevention. However, few longitudinal studies of the relationship between these two species and caries activities in children [12, 14] or individuals with intellectual disabilities (ID) [15] and Down syndrome [16] have been performed.

Individuals with ID that are cared for at home by family members may also suffer from poor oral hygiene, as higher bacterial counts were found in subjects with ID as compared to those with no disabilities [17]. The methods available to treat and prevent dental caries, and improve oral hygiene individuals with severe disabilities are limited. However, it is important for clinicians to choose from among the various techniques available for prevention of dental caries in their patients with ID according to risk. In the present study, we detected *S. mutans* and *S. sobrinus* in Japanese patients with ID using a PCR method, and then compared their presence with the incidence of dental caries over a 1-year period.

## Methods

One hundred forty-five outpatients with ID (an Intelligence Quotient (IQ) ≤ 70) aged 6 to 30 years and each with mixed or permanent dentition, who visited Hiroshima University Hospital, Hiroshima City were enrolled. The demographic details of the participating outpatients and data on their disability status, IQ, systemic diseases and history of regular medications (if any) were collected from the medical records. Subjects were comprised of 135 with ID and 10 with Down syndrome, and a total of 106 (73.1 %) males and 39 (26.9 %) females were examined (Table 1). Consent for participation was obtained from at least one of their parents prior to the study according to the ethical guidelines of the Declaration of Helsinki (1975) and ethical clearance was obtained from the Ethical Committee of Hiroshima University (Epidemiology-No. 34). Each subject underwent a dental examination performed in the Special Care Dental Clinic by a single well-trained dentist (M.O.) while seated in a dental chair in a supine position, using the WHO caries diagnostic criteria to determine the decayed and filled teeth (DFT) index [18]. Those who had received antibiotics within the previous 3 months or with systemic diseases were excluded.

**Table 1:** Study population by age and sex

Age	Male		Female		Total
(years)	No.	%	No.	%	
6–10	12	92.3	1	7.7	13
11–15	19	86.4	3	13.6	22
16–20	21	77.8	6	22.2	27
21–25	32	69.6	14	30.4	46
26–30	24	64.9	13	35.1	37

Dental plaque was collected from all erupted teeth by brushing with a sterile toothbrush for 1 min using a previously described method [19]. During toothbrushing, plaque adhering to the toothbrush was removed by washing several times in a tube of sterile distilled water. The plaque samples in the tube were immediately transported to our research laboratory and stored at −20 °C, prior to extraction of genomic DNA.

*Streptococcus mutans* JCM5175 and *S. sobrinus* ATCC27607 were used as control species. PCR detection of the target species was performed using primers described by Igarashi et al. [8, 10]. Oligonucleotide primers were designed to the *dex* DNA sequence of *S. mutans* (GenBank accession no. D49430) and *S. sobrinus* (GenBank accession no. M96978). For *S. mutans*, the forward primer, 5’ TAT GCT GCT ATT GGA GGT TC 3’, is complementary to the sequence 973–992, and the reversed primer, 5’ AAG GTT GAG CAA TTG AAT CG 3’, is complementary to the sequence 2225–2244. For *S. sobrinus*, the forward primer, 5’ TGC TAT CTT TCC CTA GCA TG 3’, is complementary to the sequence 134–153, and the reversed primer, 5’ GGT ATT CGG TTT GAC TGC 3’, is complementary to the sequence 1726–1743.

The primers for eubacteria 16S ribosomal RNA sequence (GenBank accession number M75035) were used to confirm the presence of bacteria in plaque samples as positive control [20]. The forward primer, 5’ CAG GAT TAG ATA CCC TGG TAG TCC ACG C 3’, is complementary to the sequence 783–810, and the reversed primer, 5’ GAC GGG CGG TGT GTA CAA GGC CCG GGA ACG 3’, is complementary to the sequence 1378–1407. The size of the expected PCR product was 625 bp.

Plaque samples were first subjected to centrifugation at 1600 × *g* for 20 min. Next, supernatant was discarded, and individual cell pellets were stored at −20 °C until DNA isolation, for which the pellets were resuspended in 180 μl of enzymatic lysis buffer (20 mM Tris–HCl, pH 8.0; 2 mM EDTA; 1.2 % Triton X-100, 20 mg/ml lysozyme). A genomic DNA preparation from each plaque sample was obtained using a DNeasy® Blood and Tissue Kit (Qiagen, Austin, TX, USA) for DNA extraction of Gram-positive bacteria, to which we added an RNase treatment [21]. DNA concentrations in the dental plaque samples were determined by measuring *A*_260_, while quality was estimated using the *A*_260_/*A*_280_ ratio [22].

Polymerase chain reaction amplification was performed in a reaction mixture (25 μl) consisting of PCR beads (GE Healthcare UK Limited, Little Chalfont, Buckinghamshire, UK) that contained an enzyme (two units of *Taq* DNA polymerase) and the required reagents, as well as 25 pmol of each primer, and 20 to 50 ng of the template DNA solution in a thermal cycler (DNA Engine PTC-220 DYAD^TM^, MJ Research, Hatoboro, PA, USA). Each set of PCR analyses included a negative control (water blank) in addition to the positive control. The reaction mixture was denatured at 95 °C for 3 min, followed by 26 cycles of denaturation at 95 °C for 1 min, annealing at 55 °C for 1 min, and extension at 72 °C for 1 min, with a final cycle of 94 °C for 1 min, 55 °C for 1 min, and 72 °C for 5 min [8]. Following amplification, 15 μl of the PCR products was analyzed by electrophoresis on a 1.0 % agarose gel and, after staining with ethidium bromide, the newly synthesized DNA fragments were visualized under ultraviolet light at 302 nm. The sizes of the PCR products were estimated from the electrophoretic migration of products relative to a 100-base ladder marker (Amersham Pharmacia Biotech, AB, Uppsala, Sweden). The sensitivity of the PCR method was tested by using known amounts of purified *S. mutans* JCM5175 and *S. sobrinus* ATCC27607 as a template. Serially diluted DNA was also used as a template, and the detection limit was 1 pg of DNA for *S. mutans* (12 cells) and 100 fg of template DNA for *S. sobrinus* (9 cells) (data not shown).

Descriptive statistics and statistical analyses were performed using a software statistical package (SPSS 14.0, Inc., Chicago, IL, USA). A Mann–Whitney U-test was employed to compare the caries scores between subjects possessing the various combinations of bacteria, and a Wilcoxon rank test to compare caries scores between the baseline and 1 year later.

## Results

Table 2 shows caries prevalence in subjects with ID possessing *S. mutans* alone or in combination with *S. sobrinus* at baseline and after 1 year. *S. mutans* and *S. sobrinus* were found in 78.7 and 83.5 %, respectively, of our subjects with ID. In addition, 13.1 % were positive for *S. mutans* alone, 17.9 % for *S. sobrinus* alone, and 65.6 % for both, while 3.4 % were negative for both *S. mutans* and *S. sobrinus*. The DFT score in those positive for both organisms after 1 year was significantly higher than that of those positive for *S. mutans* alone (*P* < 0.01). Furthermore, that score in subjects positive for both after 1 year was significantly higher than at baseline (*P* < 0.001) and the increase of DFT score in those positive for both was significantly greater than in subjects positive for *S. mutans* alone (*P* < 0.01).

Table 3 shows subjects with previous caries experience at baseline and caries incremental increases after 1 year, along with the combination of mutans streptococci detected. Seventeen (89.5 %) with *S. mutans* alone, and 86 (90.5 %) with both *S. mutans* and *S. sobrinus* had past caries experiences. Four (21.1 %) of the subjects with *S. mutans* alone, 9 (34.6 %) with *S. sobrinus* alone, and 54 (55.7 %) with both had increases in caries increment, while none of the subjects possessing neither organism showed an increase in increment. Also, approximately 60 % of the subjects with both had an increase in increment caries over the 1-year study period, whereas approximately 20 % of those with *S. mutans* alone demonstrated such an increase.

**Table 2:** Caries prevalence in patients with *S. mutans* alone or in combination with *S. sobrinus* at baseline and after 1 year

Oraganism present			Mean (SD) DFT		Increase in DFT	Range
*S. mutans*	*S. sobrinus*	No. of subjects (%)	Baseline	After 1 year	Mean (SD)	
+	-	19 (13.1)	6.47 (4.64)	6.95 (4.61)*	0.47 (1.22)*	0–5
-	+	26 (17.9)	5.96 (5.03)	6.42 (5.33)	0.46 (0.71)	0-2
+	+	95 (65.6)	8.65 (7.60)†	10.31 (8.13)* †	1.65 (2.04)*	0–9
-	-	5 (3.4)	0.60 (1.34)	0.60 (1.34)	0.00 (0.00)	0

Statistical significance between groups: *P<0.01, Mann–Whitney U-test; †*P*<0.001, Wilcoxon rank test.

Data for DFT scores are presented as mean (SD)

**Table 3:** Patients with previous caries experience at baseline and incremental increases after 1 year, and the combination of mutans streptococci detected

Organism present		Previous caries experience		Caries incremental increase	
*S. mutans*	*S. sobrinus*	No.	%	No.	%
+	-	17	89.5	4	21.1
-	+	22	84.6	9	34.6
+	+	86	90.5	54	55.7
-	-	1	20.0	0	0.0

PCR analysis with 16S rRNA primers confirmed the presence of bacterial DNA in all plaque samples (data not shown).

## Discussion

Individuals who are intellectually disabled and cared for at home by family members often suffer from poor oral hygiene, as they have been reported to have higher bacterial counts as compared to individuals with no disabilities [17]. The methods available to treat and prevent dental caries, and also improve oral hygiene for patients with severe disabilities are limited, though it is important for clinicians to choose a suitable technique for prevention of dental caries based on the risk of their development in their patients with ID.

We performed the present longitudinal study to compare the presence of mutans streptococci with the incidence of dental caries in Japanese patients with ID over a 1-year period using a PCR method. The average concentration of DNA recovered from brushing plaque samples was approximately 700 μg/ml, which was deemed sufficient for performing this PCR-based survey. To ensure presence of a representative bacterial sample in all cases and absence of PCR inhibiting substances, we performed a broad-range PCR assays applying eubacterial 16S rRNA-based primers and subjecting all samples obtained by the toothbrushing method. This confirmed the presence of bacteria and bacterial DNA in all plaque samples (data not shown). This method has been shown to be a more sensitive means of detection of cariogenic microorganisms as compared with conventional culture techniques [8–11].

The present study results indicate that the prevalence of mutans streptococci in patients with ID aged 6 to 30 years old is 96.6 %, which is in agreement with similar surveys conducted with school children in other parts of the world [23–26], while the prevalence of mutans streptococci in adults has been reported to be greater than 60 % [27] and 82.7 % in subjects with Down syndrome aged 1–48 years old [16]. The prevalence of *S. sobrinus* alone in the present study was higher than in those studies. However, those studies employed cultural methods and previous findings have shown that mitis-salivarius bacitracin agar inhibits the growth of *S. sobrinus* to a greater degree than that of *S. mutans* [28, 29]. Thus, the contrasting results may have been due to the more sensitive isolation and detection method used for *S. sobrinus* in our study as compared to those employed in the past, as well as differences in type and stage of carious lesions in the subjects. Nevertheless, since the present cohort was limited, additional studies are required.

The DFT score in those positive for both organisms at after 1 year was significantly higher than that of those positive for *S. mutans* alone. Furthermore, that score in subjects positive for both after 1 year was significantly higher than at baseline and the increase of DFT score in those positive for both was significantly greater than in subjects positive for *S. mutans* alone. The caries incremental increase for subjects possessing both organisms was 3.5 times higher than that for those with *S. mutans* alone, indicating that individuals with ID possessing both have higher caries activities that those with only *S. mutans*. In our previous study, the increase in dft score for primary teeth in preschool children possessing both was four times greater than for those possessing *S. mutans* alone [12]. The present PCR results showed that subjects with ID possessing both *S. mutans* and *S. sobrinus* had a significantly higher caries incidence and increment increase as compared to those with only *S. mutans* at after 1 year, which also agrees with the results of previous studies of schoolchildren aged 11–12 years old [30, 31], as well as studies of preschool children aged 3–5 years old [6, 12, 32]. In contrast, there was no significant difference in baseline score for DFT between subjects positive for both *S. mutans* and *S. sobrinus* and those with *S. mutans* alone, though the DFT score of those positive for both *S. mutans* and *S. sobrinus* showed a trend to be higher than that of those with *S. mutans* alone.

The present subjects were being treated as outpatients and were referred from local dental clinics because of their poor dental cooperation and already had untreated decayed teeth. All decayed teeth were immediately treated in the Special Care Dental Clinic, thus the caries incremental increase in subjects positive with both *S. mutans* and *S. sobrinus* was significantly higher than in those with *S. mutans* alone. In previous studies, subjects who possessed both bacteria had increases in caries increment over a 1-year period [14, 33]. The results of that former study suggested that the prevalence of *S. sobrinus* was more closely associated with future caries activity, especially with an increase in smooth-surface caries increment, than the prevalence of *S. mutans*. However, the subject population in the present study was limited, thus further studies are required. In addition, it remains unknown how mixed colonization increases the cariogenicity of bacteria, while quantitative studies using a real-time quantitative PCR are required to understand the mechanisms of increased caries risk for future studies.

Seventeen (89.5 %) with *S. mutans* alone, and 86 (90.5 %) with both *S. mutans* and *S. sobrinus* had past caries experiences. Four (21.1 %) of the subjects with *S. mutans* alone, 9 (34.6 %) with *S. sobrinus* alone, and 54 (55.7 %) with both had increases in caries increment, while none of the subjects possessing neither organism showed an increase in increment. Also, approximately 60 % of the subjects with both had a caries incremental increase over the 1-year study period, whereas approximately 20 % of those with *S. mutans* alone demonstrated such an increase.

## Conclusions

In conclusion, the present longitudinal study results indicate that individuals with ID harboring both *S. mutans* and *S. sobrinus* have a significantly higher incidence of dental caries than those positive for *S. mutans* alone.
